# Ultrasonographic Evaluation of Fatty Pancreas in Serbian Patients with Non Alcoholic Fatty Liver Disease—A Cross Sectional Study

**DOI:** 10.3390/medicina55100697

**Published:** 2019-10-17

**Authors:** Tamara Milovanovic, Sanja Dragasevic, Milica Stojkovic Lalosevic, Sanja Zgradic, Biljana Milicic, Igor Dumic, Stefan Kmezic, Dusan Saponjski, Andrija Antic, Velimir Markovic, Dragan Popovic

**Affiliations:** 1 School of Medicine, University of Belgrade, 11 000 Belgrade, Serbia; tamara.alempijevic@med.bg.ac.rs (T.M.); drmilicastojkovic@gmail.com (M.S.L.); drandrija.antic@gmail.com (A.A.); mbecambeca@yahoo.com (V.M.); dragan.drendo23@gmail.com (D.P.); 2 Clinic for Gastroenterology and Hepatology, Clinical Center of Serbia, 11 000 Belgrade, Serbia; dragasevicsanja@gmail.com (S.D.); sanjazgradic@gmail.com (S.Z.); 3 Institute for Medical Informatics and Biostatistics, School of Dentistry, University of Belgrade, 11 000 Belgrade, Serbia; biljana.milicic@sbb.rs; 4 Division of Hospital Medicine, Mayo Clinic Health System, Eau Claire, WI 54703, USA; igordumic84@gmail.com; 5 Mayo Clinic College of Medicine and Sciences, Rochester, MN 55905, USA; 6 Clinic for Abdominal Surgery—First Surgical Clinic, Clinical Center of Serbia, 11 000 Belgrade, Serbia; kstefan1986@gmail.com; 7 Center for Radiology and MRI, Clinical Center of Serbia, 11 000 Belgrade, Serbia

**Keywords:** pancreas, non–alcoholic fatty liver disease, metabolic syndrome, prediction, scoring system

## Abstract

*Background and Objectives*: The aim of the study was to determine the association between presences of fatty pancreas (FP) with the features of metabolic syndrome (MeS) in patients with non–alcoholic fatty liver disease (NAFLD) and to establish a new noninvasive scoring system for the prediction of FP in patients with NAFLD. *Material and Methods:* 143 patients with NAFLD were classified according to FP severity grade into the two groups and evaluated for diagnostic criteria of MeS. All patients underwent sonographic examination with adiposity measurements and the liver biopsy. Liver fibrosis was evaluated semi-quantitatively according to the METAVIR scoring system and using non-invasive markers of hepatic fibrosis. *Results:* Waist circumference (WC) was predictive for increased risk of FP in NAFLD patients. Elevated fasting plasma glucose, total cholesterol, serum amylase and lipase levels were associated with presence of severe FP (*p* value = 0.052, *p* value = 0.007, *p* value = 0.014; *p* value = 0.024, respectively). Presence of increased amounts of mesenteric fat was associated with severe FP (*p* value = 0.013). The results of this study demonstrated highly significant association between NAFLD and presence of FP. The model for predicting the presence of FP was designed with probability value above 6.5. *Conclusion:* Pancreatic fat accumulation leads to worsening of pancreatic function which in turns exacerbates severity of metabolic syndrome associated with both, NAFLD and NAFPD.

## 1. Introduction

Obesity as a growing public health problem worldwide, is often associated with elevated fasting glucose, dyslipidemia and hypertension-common disorder defined as metabolic syndrome (MeS) [1,2]. Nevertheless, up to 20% of obese people have no insulin resistance, disorders in carbohydrate metabolism or any of the other conditions associated with obesity, which highlights heterogeneous and complex pathophysiological background of MeS [3].

The prevalence of MeS varies by geographic region, ethnicity and gender. It increases with age in sex-specific manner and affects over 30% of the adult population in the United States, China and Europe [3,4]. The age-related increase in prevalence of MeS in women occurs as the result of sex-related factors associated with hyperandrogenism, insulin-resistance and menopause [3]. In Europe, prevalence of MeS has been increasing across all age groups and in both genders, with a 5-fold increase in women and a 2-fold increase in men [4]. According to The International Diabetes Federation and Adult Treatment Panel (ATP III), diagnostic criteria for MeS includes central obesity, defined by specific waist circumference (WC) beyond the ethnic standard and the presence of two or more of the following: impaired glucose tolerance, dyslipidemia and high blood pressure [5,6]. In MeS, insulin resistance, triggers hyperglycemia and hepatic lipid synthesis, which consequently leads to accumulation of tissue fat [1].

Development of certain digestive diseases with abnormal fat accumulation, has been associated with endocrinologically active, visceral adipose tissue, increased oxidative stress and increased production of adipokines, cytokines and monocyte chemotactic protein-1, which promote low-grade inflammation and accelerate the migration of the bone marrow-derived monocytes/macrophages and tissue homing [1,7].

The relation of non-alcoholic fatty liver disease (NAFLD) and MeS has been previously described with a special focus for diabetes mellitus development in these patients. Non–alcoholic fatty liver disease (NAFLD) can range from simple steatosis, non-alcoholic steatohepatitis, cirrhosis and end stage liver disease [8,9].

Non-alcoholic fatty pancreas disease (NAFPD) or fatty pancreas (FP) is a disorder with excessive lipid accumulation within the pancreas in the absence of alcohol intake [7,8,10]. In FP, accumulated fat leads to inflammation within pancreatic islet cells likely caused by presence of 12/15 lipoxygenase. It affects β cell function by suppression of antioxidant enzymes activity [8]. Furthermore, the endocrine and exocrine pancreas dysfunction can be associated with direct toxic effect of accumulated fat in pancreatic acinar cells that might lead to development of pancreatic cancer [8].

The clinical implications of NAFPD are still controversial and the topic of ongoing debate. The association between increased body mass index (BMI), insulin resistance and fatty liver disease has been previously reported [8,11]. It has been shown that fatty infiltration of pancreas correlates with presence of metabolic risk factors and may represent another significant manifestation of MeS [11]. Therefore, the aim of this study was to determine the association between presences of FP in our NAFLD patient cohort with features of MeS. Additionally, we aimed to establish a novel, simple and noninvasive scoring system for prediction of presence of FP in patients with NAFLD.

## 2. Material and Methods

### 2.1. Recruitment of Participants

We conducted an observational, cross-sectional study at the Clinic for Gastroenterology and Hepatology, Clinical Center of Serbia, Belgrade that included 143 patients with NAFLD. Exclusion criteria were the following: age < 18 years, presence of any other chronic liver disease (CLD), alcohol consumption, presence of hepatocellular carcinoma or severe chronic extra-hepatic disease, admission due to other chronic illness, and human immunodeficiency virus infection.

The liver biopsy was performed on each patient in order to evaluate liver fibrosis according to the METAVIR scoring system. In accordance with histopathological analysis, patients were categorized into the two groups: mild/moderate or advanced fibrosis. A detailed description of our research study methods is described in the Liver biopsy section.

All patients were further classified, based on severity grade of FP, measured by ultrasonography, into the two groups: the first group included patients with non-fatty pancreas and grade I (mild) FP and the second group included patients with grade II (moderate) and grade III (severe) FP [12].

All patients were screened for diagnostic criteria of metabolic syndrome proposed by the Adult Treatment Panel (ATP) III and The International Diabetes Federation [5,7] (Table 1). Clinical data and review of dietary, smoking and exercise habits were included in the analysis. The data pertinent to parameters of metabolic syndrome were collected from medical records and routine laboratory testing which included: complete blood cell count, biochemistry, fasting plasma glucose (FPG) level, triglycerides (TG), total cholesterol, low-density lipoprotein (LDL) cholesterol and high-density lipoprotein (HDL) cholesterol level. Anthropometric measurements (weight, height and waist circumference), age and sex were obtained and body mass index (BMI) was calculated. Male patients with WC ≥ 90 cm and women with WC ≥ 80 cm were diagnosed with central obesity. BMI was calculated by dividing weight in kilograms by square of height in meters, with values of BMI ≥ 25 kg/m^2^ defined as obese. Hypertension was defined as systolic blood pressure ≥ 18.6 kPa or diastolic blood pressure ≥ 11.9 kPa. Type II diabetes mellitus was defined according to recommendations from the American Diabetes Association [13].

Homeostatic Model Assessment of Insulin Resistance (HOMA-IR) was used to evaluate the presence and the extent of insulin resistance. It was calculated according to the following formula: fasting insulin level (mU/L) × fasting glucose level (mg/dL). In healthy adults normal range is between 0.5–1.4, with optimal insulin sensitivity < 1.0. Early insulin resistance was defined by values > 1.9 and values above 2.9 indicated significant insulin resistance.

Simple method for Quantifying Metabolic Syndrome (SiMS) score was used as a developed method for quantification of metabolic status and calculated using the following formula: 2*Waist/Height + Gly/5.6 + Tg/1.7 + TAsystolic/130—HDL/1.02 or 1.28 (for male or female subjects, respectively) [14].

### 2.2. Abdominal Ultrasound Examination

All patients included in the study underwent abdominal ultrasound (US) examination with high resolution ultrasonography using a 3.5 MHz linear transducer and standard approach. Fatty pancreas was diagnosed in cases of increased echogenicity of the pancreas body compared with that of liver at the same depth on a longitudinal scan near the abdominal line, or with the renal cortex when liver showed increased echogenicity. All subjects were categorized into the following four groups: non fatty pancreas, grade I or mild FP when pancreas echogenicity was higher than kidney’s, but lower than echogenicity of retroperitoneal fat, grade II or moderate FP when pancreas echogenicity was higher than kidney but lower than echogenicity of retroperitoneal fat and grade III or severe FP when pancreas echogenicity was similar to that of retroperitoneal fat.

Sonographic measurements of adipose tissue were performed including the thickness of perirenal fat (PRFT), and mesenteric fat tissue (MAFT) [15]. The ultrasound PRFT was measured from the inner side of the abdominal musculature to the surface of the kidney [15,16]. The measurement of MAFT was performed with special attention to the identification of the mesenteric leaves appearing as elongated structures—with highly reflective peritoneal surfaces. Measurements of three thickest mesenteric leaves were made on each ultrasonographic examination and the mean was used for further analysis [17,18].

### 2.3. Liver Biopsy

Percutaneous liver biopsy was performed on each patient, and specimens were routinely processed. The single experienced pathologist blinded for patients’ clinical data and the results of noninvasive methods analyzed sections independently [18]. Liver fibrosis was evaluated semi quantitatively according to the METAVIR scoring system. Fibrosis was scored on a scale of 0–4, as following: F0 = no fibrosis, F1 = perisinusoidal/periportal fibrosis, F2 = portal fibrosis and few septa, F3 = numerous septa without cirrhosis, F4 = cirrhosis. Stages F0, F1 and F2 were considered as mild/moderate fibrosis, while F3 and F4 were considered as advanced fibrosis. According to histopathological analysis, patients were categorized into the two groups: mild/moderate or advanced fibrosis.

### 2.4. NAFLD Non-Invasive Markers of Hepatic Fibrosis

NAFLD fibrosis score (NFS), BARD score, Fibrosis-4 score (FIB-4) and AST to Platelet Ratio Index (APRI) were used for noninvasive assessment of fibrosis in non-alcoholic fatty liver disease [19]. Table 2 shows the selected scores and their equations. NAFLD fibrosis score was based on clinical parameters with score below 1.455 indicating negative predictive value for exclusion of advanced fibrosis. BARD score was calculated with values ≥ 2 suggestive of advanced fibrosis. FIB-4 index values below 1.5 indicated negative predictive value for presence of advanced fibrosis. Values of APRI greater than 0.7 were identified as marker predictive of significant hepatic fibrosis [19].

### 2.5. Data Analysis

Statistical analysis was performed using the statistical software package SPSS 23.0 for Windows (SPSS, Inc., Chicago, IL, USA). Descriptive data for all groups and variables were expressed as mean ± SD for continuous measures, or percent of a group for discrete measures. Categorical data were analyzed using the Pearson chi-square test. Normal distribution was tested using the Kolmogorov-Smirnov test. If the data were normally distributed, the t-test was used. Non-parametric data were analyzed using the Mann Whitney U test. Logistic regression model was used to determine predictors of different grades of FP. ROC curve was used to determine the validity of the obtained score. The *p* value < 0.05 was required to reject the null hypothesis and considered statistically significant.

### 2.6. Ethical Considerations

This study was conducted following the approval from The Ethic Committee of Clinical Centre of Serbia numbered 415/6 (approval date 21/09/2017) and in accordance with the Helsinki Declaration. All patients provided written informed consent before inclusion in the study.

## 3. Results

### 3.1. Clinical and Laboratory Characteristics of Patients in Association with Grade of FP

Selected demographic and clinical patients’ characteristics associated with presence of fatty pancreas are showed in Table 3 (patients with grade 0 and 1 are merged into Grade I FP group, and with grade 2 and 3 into Grade II FP group). Comparing the two groups of patients, the final analysis showed no significant differences in demographic variables, BMI, systolic and diastolic blood pressure, presence of hypertension, dyslipidemia or statin use. In addition, no significant differences were detected in the lifestyle factors between the two groups, such as current smoking and coffee consumption.

However, the analysis demonstrated significant differences among FP groups in regard to the waist circumference and identified WC as a prognostic factor for higher risk of development of FP in patients with NAFLD (*p* value = 0.018). We observed that diabetes mellitus (DM) was identified in 39 patients with NAFLD, with insulin independent sub-type being more frequent in patients with moderate and severe FP (*p* value = 0.02 and *p* value = 0.01, respectively). Our results demonstrated a statistically significant difference in values of SiMS score among the study groups, with higher values observed in patients with severe FP (*p* value = 0.02).

As shown in Table 4, there was no significant difference in the values of insulin level, HDL and triglycerides in the two groups. Nevertheless, higher values of fasting plasma glucose, total cholesterol, serum amylase and lipase were associated with the presence of moderate and severe fatty pancreas (*p* value = 0.052, *p* value = 0.007, *p* value = 0.014 and *p* value = 0.024, respectively). Furthermore, our results demonstrated a greater proportion of patients with severe FP in those with HOMA-IR > 3, but the statistical analysis revealed no significant association. Out of all 39 NAFLD patients with DM, 33 had values of HbA1c > 6%. According to our results, HbA1c values higher than 6 were significantly associated with the presence of severe fatty pancreas, highlighting its impaired function.

### 3.2. Association between Presence of FP and the Use of Antidiabetic Agents, Statins and Antihypertensives

No significant difference was found in the use of statins and anti-hypertensive agents among the two groups of patients (Table 5). We observed that higher number of patients with severe FP was not on antidiabetic therapy. Patients who were treated with metformin and glimepiride were more likely to have mild FP (*p* value = 0.035) (Table 5).

### 3.3. Association between Presence of FP and Ultrasonographic Measurements of Visceral Fat

We demonstrated significant difference among FP groups in regards to amounts of visceral and mesenteric fat, with higher values of both associated with group who had severe FP (*p* value = 0.013). However, there was no significant difference in amounts of perirenal fat between the two groups (*p* value = 0.847). In addition, no significant difference was registered in pancreatic body thickness among FP groups in our cohort of patients with NAFLD (*p* value = 0.412) (Table 6).

### 3.4. Association between FP and Non-Alcoholic Fatty Liver Disease

As shown in Table 7, the results of our study showed highly significant association between NAFLD and the presence of fatty pancreas (*p* value = 0.04).

According to histological analysis, 130 (90.9%) patients had mild/moderate fibrosis and 13 (9.1%) had advanced fibrosis. However, there was no association between presence of FP in regards to histopathological grade of liver fibrosis (*p* value = 0.830).

NAFLD fibrosis index was in all cases higher than 1.455. Table 8 shows the association between FP and non-invasive markers of fibrosis in NAFLD patients (BARD score, APRI and FIB4). Our results did not find significant association of these score values with the presence of fatty pancreas (*p* value = 0.775, *p* value = 0.326 and *p* value = 0.961, respectively).

### 3.5. Factors Related to Diagnosis of Non-Alcoholic Fatty Pancreas Disease

In the multivariate analysis of FP predictors in our study cohort, we used the logistic regression approach. In the logistic regression model, we used patients’ data on levels of fasting glucose, total cholesterol, serum amylase, lipase and hepatic steatosis, since these factors showed significant association with presence of FP at the univariate analysis.

**Figure 1:**
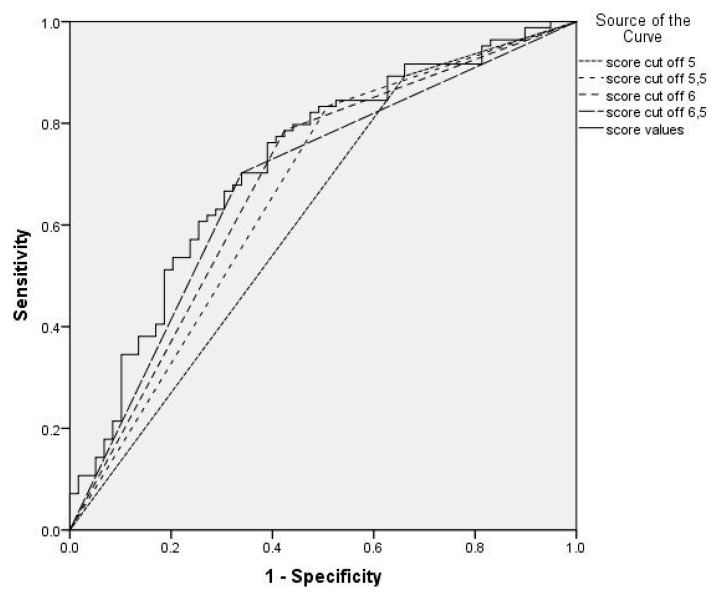
ROC curve illustrates changes in sensitivity and specificity of the score for prediction of FP in NAFLD patients relative to different cut off points.

Model of predicting occurrence of FP was derived from multivariate logistic regression analysis. The probability was estimated using the following equation: 0.627 + 0.640 * 0.593 * glucose (fasting glucose level)—cholesterol level (total cholesterol) + 0.058 + 1.585 * serum lipase * ultrasonography level of liver steatosis (Table 9). According to the score values for different cut off levels, best ability to predict presence of severe FP has been shown for the score value above 6.5. (Table 10). Figure 1 presents ROC curve for different cut off values of this score.

## 4. Discussion

Pancreatic steatosis has been first described in postmortem studies from 1978 and 1984. These studies demonstrated an association between FP and age, BMI, generalized atherosclerosis, diabetes and pancreatic fibrosis [20,21].

Published data on NAFPD and its clinical implications highlighted its association with metabolic risk factors, insulin resistance and hepatic steatosis [7,8,10]. It has been suggested that FP is a risk factor for development of MeS or another manifestation of it [10,11].

In this study, we report data from 143 patients with NAFLD in whom we analyzed possible association with excessive lipid deposition in pancreas and features of MeS, introducing the new score for predicting occurrence of FP in this group of patients.

To date, literature on FP is lacking consistent reports. Lee JS et al. described both, FP and NAFLD to be associated with visceral fat and insulin resistance, suggesting that FP can be an initial indicator of ectopic fat deposition and present an early marker of insulin resistance which is a hallmark of MeS [22]. NAFPD has been associated with increased BMI and HOMA-IR, however no significant association was detected with fasting glucose levels [22]. Uygun et al. investigated the association between FP and serum glucose levels in patients with NASH. They found no statistical difference in insulin levels and HOMA-IR among the groups, but demonstrated significantly higher HbA1c in the group of patients with NASH who also had FP [9]. Ou et al. reported increase in glucose levels in NAFLD patients with FP and found similar trends for hypertension, obesity, HDL level and hypertriglyceridemia. The same study also demonstrated that NAFLD and FP were also associated with presence of diabetes [10].

Our investigation subdivided NAFLD patients by the degree of pancreas echogenicity in two groups. It failed to demonstrate difference in regards to demographic data, BMI, insulin levels, HDL and triglyceride levels. However, we found significant difference in the waist circumference between the two groups. We conclude that WC can predict presence of severe FP in patients with NAFLD. Furthermore, higher values of fasting glucose, total cholesterol, serum amylase and lipase were associated with presence of moderate and severe FP. There was no significant association between HOMA IR and severity of FP. Our results demonstrated that HbA1c values higher than 6 were associated with presence of severe FP in patients with NAFLD. It implies impaired pancreatic function in patients who have MeS. Similar to Ou et al., results of our study demonstrated the association between DM with moderate to severe FP in patients with NAFLD.

Previous studies have indicated that fatty infiltration of pancreas leads to loss of β-cell’s mass and function, contributing to the development of DM [10]. Additionally, obesity and NAFLD are associated with impaired glucose metabolism. In contrast, results of some studies found no relation between pancreatic fat content and β-cell function in patients with impaired glucose metabolism [10,23].

Interestingly, results of our study showed that the high number of patients with severe FP was not on treatment with antidiabetic agents. Use of metformin and glimepiride was associated with presence of mild FP. Keeping SiMS in mind the results of this study, we argue that the use of antidiabetic agents in the patients with NAFLD might have protective and ameliorating effect on MeS, hence, they might be indicated in these patients.

Although the results of our study showed no significant difference in the presence of hypertension and dyslipidemia, the values of SiMS score showed significant association with severity of FP and in our patient cohort the higher values of SiMS score were found in patients with severe FP. This finding emphasize an important connection between metabolic syndromes in the development of FP.

Several recently conducted studies suggested that measuring visceral fat thickness by ultrasonography is an effective screening tool for metabolic syndrome and estimation of the risk for development of NAFLD [15,16,17]. Lirussi Fet al. concluded that perihepatic adipose tissue thickness may represent a noninvasive marker for predicting severity of liver damage in NAFLD patients [16]. Additionally, Liu KH et al. introduced increased mesenteric fat thickness as a risk factor for development of fatty liver independent of BMI, age, sex, insulin resistance, blood pressure, fasting plasma glucose or lipid levels with odds ratio of 1.5 for every 1 mm increase in MAFT [17]. We analyzed the relationship between visceral fat thickness and presence of FP in our patient cohort. The results showed significant difference in the thickness of mesenteric fat among FP groups with higher values found in the group who had severe fatty pancreas.

Non-alcoholic fatty liver disease (NAFLD) presents a spectrum of disorders ranging from simple steatosis and steatohepatitis to fibrosis and cirrhosis [24]. Liver biopsy is a gold standard for diagnosis of liver cirrhosis, however due to its invasive nature noninvasive scoring systems were developed for assessment of fibrosis in patients with NAFLD [25,26]. Rath MM et al. concluded that noninvasive scoring systems like NFS, BARD and APRI are not sensitive enough to detect fibrosis, but highly specific to include it for scores higher then specific cut offs [25]. Van Greenen EJ et al. conducted a postmortem study on 80 cadavers and found that total pancreatic fat is a significant predictor for presence of NAFLD, but there was no correlation between pancreatic fat and NAFLD activity score [27]. The results of the study by Lesama CR et al. confirmed the association between NAFLD and NAFPD and their correlation with metabolic risk factors [28]. Results of our study did not demonstrate an association between the severity of FP and values of non-invasive markers commonly used to differentiate between mild, moderate and advanced liver fibrosis in patients with NAFLD.

The ultrasound is the most widely used imaging modality for pancreas evaluation. It defines pancreatic steatosis as higher pancreatic echogenicity in comparison to liver or kidney. Pancreas echogenicity similar to the one of retroperitoneal fat suggests the highest level of fat deposition within it [29]. In their study, Al-Haddad et al. reported that presence of fatty liver is a predictor for presence of FP as seen at endoscopic ultrasound with nearly 14-fold odds ratio [30]. Patel NS et al. also reported the association between increased pancreatic fat and hepatic steatosis in patients with NAFLD who underwent MRI; however, liver fibrosis was inversely associated with FP [31]. Results of our study showed highly significant association between NAFLD and the presence of fatty pancreas. However, no association was found between the presence of FP and histopathological grade of liver fibrosis.

Recent changes in definition of MeS, different criteria for clinical diagnosis as well as recently recognized association between NAFLD and NAFPD created a possibility for new approach in assessment of presence of FP in NAFLD. Using multivariate logistic regression analysis, we created a new score for predicting occurrence of FP among NAFLD patients with the ability to predict severe FP at values above 6.5.

## 5. Conclusions

Pancreatic fat accumulation was associated with worsening of pancreatic function and other metabolic syndromes. Metabolic syndrome is associated with both, NAFLD and NAFPD. In this study we found strong correlation between these two conditions and it is likely that fatty pancreas might be one of the first manifestation of metabolic syndrome. Presence of FP is associated with further progression of metabolic syndrome and its consequences. Early detection of fatty pancreas might be of vital importance for early intervention, prevention and treatment of metabolic syndrome epidemic.

## Figures and Tables

**Table 1:** International Diabetes Federation and Adult Treatment Panel (ATP III) criteria for metabolic syndrome (MeS).

Criteria for Metabolic Syndrome (MeS)
Central Obesity
2 or More of Following Factors:
TG level ≥ 150 mg/dL or therapy for dyslipidemia
HDL < 40 mg/dL (males) HDL < 50 mg/dL (females) or therapy for dyslipidemia
Blood pressure (BP): systolic ≥ 17.3 kPa or diastolic ≥ 11.3 kPa or therapy for hypertension
Fasting plasma glucose FPG ≥ 100 mg/dL or previously diagnosed type 2 diabetes mellitus

Triglycerides (TG), total cholesterol, high-density lipoprotein (HDL) cholesterol level.

**Table 2:** Noninvasive markers of non–alcoholic fatty liver disease (NAFLD) used to asses fibrosis using the following equations.

Non-Invasive Markers of NAFLD	
**NFS**	−1.67 + 0.037 x age + 0.094 BMI (kg/m^2^) + 1.13 x impaired glucose tolerance/DM (yes-1; no-0) + 0.99 AST/ALT ratio-0.013 x platelets (x10^9^/L)- 0.66 x albumin(g/dL)
**BARD**	Sum of 3 variables (BMI ≥ 28—1 point; AAR ≥ 0.8—2 points; DM—1 point)
**FIB-4**	Age x AST(IU/L)
**APRI**	Platelet count(10^9^) x √ALT(IU/L) AST/AST(Upper Limit of Normal (40 IU/L)) Platelet count(10^9^)* 100

NAFLD fibrosis score (NFS), BARD score, Fibrosis-4 score (FIB-4) and AST to Platelet Ratio Index (APRI).

**Table 3:** Clinical characteristics of patients and their association with the presence of fatty pancreas (FP).

NAFLD Patients	Grade I FP	Grade II FP	*p* Value
N = 143 (%)	59 (41.3%)	84 (58.7%)	-
Age (mean ± SD)	52 ± 14	52 ± 13	0.987
Gender (male/female)	42/17	55/29	0.472
BMI (mean ± SD)	28.5 ± 4.7	29.8 ± 4.1	0.09 *
WC (mean ± SD)	101 ± 10	105 ± 11	0.018 *
Systolic blood pressure (kPa)	17.0 ± 1.3	16.9 ± 1.1	0.371
Diastolic blood pressure (kPa)	10.5 ± 1.4	10.6 ± 0.7	0.858
Hypertension (%)	31 (52.5%)	49 (58.3%)	0.492
Diabetes mellitus (DM) (%)	10 (16.9%)	29 (34.5%)	0.02 *
Insulin dependent DM (%)	6 (10.2%)	11 (13.1%)	0.595
Insulin independent DM (%)	3 (5.1%)	17 (20.2%)	0.01 *
Current smoking (%)	20 (33.9%)	19 (22.6%)	0.165
Coffee consumption (%)	40 (67.8)	61 (72.6%)	0.797
Dyslipidemia (%)	37 (62.7%)	58 (69%)	0.430
Metabolic syndrome (%)	30 (50.8%)	54 (64.3%)	0.108
SiMS score	2.9 ± 1.1	3.5 ± 1.9	0.020 *

* *p* values highlighted with * indicate statistical significance—*p* value < 0.05.

**Table 4:** Laboratory levels in patients and their association with the presence of fatty pancreas (FP).

NAFLD Patients	Grade I FP	Grade II FP	*p* Value
N = 143 (%)	59 (41.3%)	84 (58.7%)	-
Fasting plasma glucose (mg/dL)	105.7 ± 16	119.3 ± 22	0.052
Insulin level (mmol/L)	13.2 ± 4.9	15.3 ± 8.4	0.194
Hemoglobin A1c (%) 0–6	62 (43.3%)	48 (33.5%)	0.008 *
>6	10(6.9%)	23 (16.3%)	
Total cholesterol (mmol/L)	5.72 ± 1.3	6.32 ± 1.2	0.007 *
HDL (mmol/L)	1.35 ± 0.46	1.25 ± 0.38	0.153
Triglycerides (mmol/L)	1.70 ± 1.2	1.85 ± 1.5	0.20
Serum amylase	55 ± 19	69 ± 36	0.014 *
Serum lipase	33 ± 13	40 ± 18	0.024 *
HOMA-IR score (>3)	34 (57.6%)	50 (58.8%)	0.886

* *p* values highlighted with* indicate statistical significance—*p* value < 0.05.

**Table 5:** Association between presence of FP and the use of antidiabetic agents, statins and antihypertensive agents.

NAFLD Patients	Grade I FP	Grade II FP	*p* Value
*N* = 143 (%)	59 (41.3%)	84 (58.7%)	-
**Oral antidiabetic agents** No Metformin Glimepiride	56 (94.9%) 13 (15.5%) 4 (4.8%)	67 (79.8%) 2 (3.4%) 1 (1.7%)	0.035 *
**Statin use (%)**	4 (6.8%)	2 (2.4%)	0.197
**Antihypertensives**	30 (50.8%)	40 (47.6%)	0.796
No	6 (10.2%)	13 (15.5%)	
β-blockers ACE inhibitors	19 (32.2%)	23 (27.4%)	
Calcium channel blocker	3 (5.1%)	7 (8.3%)	
Polytherapy	1 (1.7%)	1 (1.2%)	

* *p* values highlighted with* indicate statistical significance—*p* value < 0.05.

**Table 6:** The association of FP and ultrasonographic measurements of visceral fat and pancreas.

NAFLD Patients	Grade I FP	Grade II FP	*p* Value
*N* = 143 (%)	59 (41.3%)	84 (58.7%)	-
Mesenteric fat thickness	15.4 ± 4.03	18.1 ± 5.9	0.013 *
Perirenal fat thickness	18 ± 6.4	18.4 ± 5.4	0.676
Pancreatic body thickness	17.09 ± 4.17	17.7 ± 4.9	0.412

* *p* values highlighted with* indicate statistical significance—*p* value < 0.05.

**Table 7:** The association between FP and NAFLD by US assessment and histopathologic analyses (HP) of liver fibrosis.

Fatty Liver	Grade I FP	Grade II FP	*p* Value
Mild steatosis	46 (32.1%)	25 (17.5%)	0.000 *
Moderate steatosis	13 (9.1%)	52 (36.4%)	0.000 *
Severe steatosis	0 (%)	7 (4.9%)	0.000 *
**Liver fibrosis (HP analysis)**	**Grade I FP**	**Grade II FP**	*p* value
Mild/moderate	54 (37.8%)	76 (53.1%)	0.830
Advanced	5 (3.5%)	8 (5.6%)	

HP—histopathologic analyses; * *p* values highlighted with * indicate statistical significance—*p* value < 0.05.

**Table 8:** The association of FP and NAFLD non-invasive markers of fibrosis.

NAFLD Markers of Fibrosis	Grade I FP	Grade II FP	*p* Value
**BARD score**			0.775
0–1	33 (23.1%)	49 (34.3%)	
2–4	26 (18.2%)	35 (24.5%)	
**APRI**			0.326
<0.7	1 (0.7%)	4 (2.8%)	
>0.7	58 (40.6%)	80 (55.9%)	
**FIB4 score**			0.961
<1.5	75 (52.4%)	44 (30.8%)	
>1.5	15 (10.5%)	9 (6.3%)	

BARD score; Fibrosis-4 score (FIB-4); AST to Platelet Ratio Index (APRI); statistical significance—*p* value < 0.05.

**Table 9:** Validation of the score in the assessment of fatty pancreas I+II vs. III+IV.

Values of the Score	AUC	Significance	Area under ROC Curve (95% CI) (AUC) *
Score	0.719	*p* = 0.000 *	0.633–0.805
Score 5	0.616	*p* = 0.018 *	0.520–0.712
Score 5.5	0.662	*p* = 0.001 *	0.569–0.756
Score 6	0.681	*p* = 0.000 *	0.592–0.772
Score 6.5	0.682	*p* = 0.000 *	0.633–0.805

* AUC—Area under the curve.

**Table 10:** Validation of different score cut off values of grade III and IV FP assessment in clinical practices.

Cut off Values	Sensitivity (95% CI)	Specificity (95% CI)	PPV (95% CI)	NPV (95% CI)
Score 5	0.893 (0.806–0.950)	0.339 (0.221–0.474)	0.658 (0.563–0.744)	0.690 (0.492–0.847)
Score 5.5	0.833 (0.736–0.906)	0.492 (0.359–0.625)	0.700 (0.600–0.788)	0.674 (0.515–0.809)
Score 6	0.786 (0.683–0.868)	0.576 (0.441- 0.704)	0.725 (0.622–0.814)	0.654 (0.509–0.780)
Score 6.5	0.702 (0.593–0.797)	0.661 (0.526–0.779)	0.747 (0.636–0.838)	0.609 (0.479–0.729)

95% CI—95% confidence interval; PPV—positive predictive value; NPV—negative predictive value.
